# Mesenchymal Stem Cells and Cancer: Clinical Challenges and Opportunities

**DOI:** 10.1155/2019/2820853

**Published:** 2019-05-08

**Authors:** Weiping Lin, Linfeng Huang, Ying Li, Bin Fang, Gang Li, Leilei Chen, Liangliang Xu

**Affiliations:** ^1^Key Laboratory of Orthopaedics & Traumatology, The First Affiliated Hospital of Guangzhou University of Chinese Medicine, The First Clinical Medical College, Guangzhou University of Chinese Medicine, Guangzhou, China; ^2^Department of Orthopaedics & Traumatology, Faculty of Medicine, The Chinese University of Hong Kong, Prince of Wales Hospital, Shatin, Hong Kong; ^3^Stem Cells and Regenerative Medicine Laboratory, Lui Che Woo Institute of Innovative Medicine, Li Ka Shing Institute of Health Sciences, The Chinese University of Hong Kong, Prince of Wales Hospital, Shatin, Hong Kong; ^4^Laboratory of Orthopaedics & Traumatology, Lingnan Medical Research Center, Guangzhou University of Chinese Medicine, Guangzhou, China

## Abstract

Stem cell-based therapies exhibit profound therapeutic potential for treating various human diseases, including cancer. Among the cell types that can be used for this purpose, mesenchymal stem cells (MSCs) are considered as promising source of stem cells in personalized cell-based therapies. The inherent tumor-tropic property of MSCs can be used to target cancer cells. Although the impacts of MSCs on tumor progression remain elusive, they have been genetically modified or engineered as targeted anticancer agents which could inhibit tumor growth by blocking different processes of tumor. In addition, there are close interactions between MSCs and cancer stem cells (CSCs). MSCs can regulate the growth of CSCs through paracrine mechanisms. This review aims to focus on the current knowledge about MSCs-based tumor therapies, the opportunities and challenges, as well as the prospective of its further clinical implications.

## 1. Introduction

MSCs are nonhematopoietic cells that were first discovered from bone marrow and reported approximately 40 years ago by Friedenstein and his coworkers [1, 2]. Studies have shown that MSCs exist in a variety of tissues. To date, MSCs have been successfully isolated from various organs including brain, liver, lung, kidney, muscle, thymus, pancreas, skin, bone marrow adipose tissue, fetal tissues, and umbilical cord [3]. Also, MSCs are known as multipotent cells which can differentiate into adipocytes, myocytes, osteocytes, and chondrocytes [4–6]. In 2006, the International Society for Cellular Therapy proposed three minimal criteria to define human MSCs. They must express CD105, CD90, and CD73 and lack expression of CD45, CD34, CD14 or CD11b, CD79*α* or CD19, and HLA-DR surface molecules. Additionally, they must adhere to plastic in culture and differentiate into osteocytes, chondrocytes, and adipocytes [7]. In addition, MSCs possess unique immunophenotypic capacity, tissue-repair capacity, and immunoregulatory capacity [8]. Therefore, owing to their relative immune evasiveness and general immune dampening activities, MSCs can be utilized in an allogenic setting and are promising seed cells for cell therapy and tissue engineering [9]. Moreover, various preclinical trials suggest that MSCs show great potential for cancer treatment, although obstacles and risks were described [10].

Studies have shown that MSCs are capable of migrating directionally to specific tissues, which is termed as homing. The tropism property of MSCs into sites of injury and tumor makes them ideal vehicles for targeted tumor therapy, although the exact mechanism of MSCs homing is not completely understood. Ongoing preclinical trials suggest that MSCs are suitable targets for cell therapy in a variety of cancers. However, the antitumor effects of MSCs are still controversial. In various types of cancer, some studies have shown proliferative effects, while others demonstrate inhibitory effects of MSCs on tumors [11]. For example, MSCs have tumoricidal effects on liver, lung cancer cell lines, and pancreatic tumors in vitro and in vivo [12–14]. In contrary, it has been shown that MSCs are capable of enhancing progression and metastasis of types of tumor, such as breast cancer and colon cancer [15–18]. In addition, MSCs may exert therapeutic function through an immune evasive mechanism, which will protect MSCs from immune detection and prolong their persistence in vivo [9]. Moreover, the survival of MSCs in the tumor and biodistribution of MSCs should take more attention when designing a trial, which may influence the results of study. For example, although human MSCs were found by staining in the tumors 1 day after IV injection in a mice model, the cells almost were cleared after 1 week [19]. However, even after 11 weeks MSCs were still observed in the tumor, although at very low numbers [19]. In an in vivo study of colon cancer, exogenous MSCs were still able to regulate immune response of the tumor microenvironment even 1 year after the last MSCs injection [20]. In this review, we summarize recent advances of MSCs in the treatment of cancer and insights into potential strategies for cancer therapy.

## 2. MSCs and Cancer

### 2.1. Discrepancy in Impacts of MSCs on Tumor Progression

Extensive studies have been performed to investigate effects of MSCs on tumor in recent decades. However, this issue is still under debate. Controversial results have been reported. Several studies have shown that MSCs promote tumor progression and metastasis through influencing signaling pathway [18, 39], while other studies suggest that MSCs affect the pathways that can suppress both proliferation and apoptosis [13, 40].

Researches have demonstrated that MSCs would be recruited into tumor sites, promoting tumor growth, and angiogenesis through differentiating into cancer-associated myofibroblasts and secretion of proangiogenic cytokines (e.g., interleukin (IL)-6, vascular endothelial growth factor (VEGF), and transforming growth factor-*β* (TGF-*β*) [21–23]. In the meanwhile, the recruited MSCs also enhanced tumor metastasis via increasing lysyl oxidase [24]. Another tumor-promoting effect of MSCs is attributed to their protection role for breast cancer cells from immune clearance through modulating regulatory T cells and inhibiting natural killer (NK) cells and cytotoxic T lymphocyte (CTL) functions [25]. Furthermore, MSCs have been found to form a cancer stem cell niche in which tumor cells can preserve the potential to proliferate and sustain the malignant process [41]. Also, increasing evidences suggest that MSCs promote tumor angiogenesis through their potential to differentiate into pericytes or endothelial-like cells as well as by their secretion of trophic factors and cytokines, proangiogenic factors, growth factors, and plasminogen activator [42, 43]. Thus, MSCs promote tumor growth and metastasis through stimulation of angiogenesis, cancer stem cell niche maintenance, and immune protection. Moreover, it has also been showed that MSCs can affect tumor development and progression through miRNAs. In a xenograft tumor model, researchers demonstrated that human umbilical cord MSCs (hUCMSCs) powerfully promote the growth of lung adenocarcinoma (LUAD) cancer cells by transferring miR-410. The findings suggest that modification of hUCMSC-derived extracellular vesicles (hUCMSC-EVs) may be a promising therapeutic option for treatment of tumor [44]. In a mice model, study found that gastric cancer tissue-derived MSCs can significantly promote HGC-27 growth and migration via increasing the expression of miR-221, which may be as a novel biomarker in gastric cancer [45]. The studies supporting MSCs favor tumor growth are summarized in Table 1.

In contrast, it has been shown that the unmodified MSCs have antitumor effects both in vitro and in different animal models of cancer, which is attributed to the factors secreted by MSCs that can suppress the proliferation of glioma, melanoma, hepatoma, and breast cancer cells [46–48]. Studies have indicated that MSCs exhibit antiglioma effect through inhibiting vascular growth in glioma cells, which is mediated by the downregulation of platelet-derived growth factor (PDGF)/PDGFR axis [28]. Also, human umbilical cord-derived MSCs (hUC-MSCs) have been shown to inhibit progression of breast cancer by inducing tumor cells death and suppressing angiogenesis [29]. Another study reported that human bone marrow-derived MSCs exhibit the potential to suppress the growth of breast cancer and inhibit lung metastasis by reducing their proliferative ability [30]. Furthermore, MSCs have been shown to have antiangiogenic effect both in vitro and in vivo [49]. MSCs also can inhibit tumor growth in a highly inflammatory and angiogenic Kaposi's sarcoma model [50]. Both in vitro and in vivo studies have shown that MSCs derived from fetal skin can inhibit the growth of human hepatocellular carcinoma (HCC) cells and can reduce cell proliferation, colony formation, and expression of oncogenes [48]. To conclude, MSCs play critical roles in processes of tumor angiogenesis, tumor immune response, and metastasis. The studies reporting MSCs inhibit tumor growth are summarized in Table 2.

### 2.2. MSCs, Cancer Stem Cells and Cancer Microenvironment

Cancer remains as one of the most challenging diseases despite extensive studies have been performed and novel systemic treatment advances during recent decades. In particular, when cancer is diagnosed to have metastasized, treatments are much less successful; while it can often be treated successfully by surgery or local irradiation before it has spread [51]. Therefore, it is necessary and imperative to better understand the biological processes behind the progression of tumor cells towards metastasis.

Cancer cells in primary tumors reside in a complex microenvironment comprising numerous cell types, including endothelial cells of blood vessels, lymphatic circulation, fibroblasts, and various bone marrow-derived cells, such as macrophages and MSCs. It has been well documented that tumor cells secret chemokines, cytokines, and growth factors recruiting MSCs into the tumor sites. In turn, MSCs, as a component of tumor microenvironment, affect tumor growth and metastasis through secretion of cytokines and chemokines [52, 53]. Thus, the process of tumor progression has been regarded as a result of an evolving crosstalk between different cell types within the tumor and its surrounding host tissue and organ or tumor stroma [54].

Cancer stem cells (CSCs), which possess chemotherapy resistance, have been considered as the root of cancers and can resist chemotherapy, explaining cancer recurrence even many years after therapy is ended. The evidence that CSCs selectively resist therapy is provided by a multitude of observations in cell culture, animal models, and cancer patients. For example, direct analysis of apoptosis during cell culture showed that differentiated colon cancer cells are induced to die after chemotherapy, while CSCs from the same culture survive after toxic damage. Moreover, these surviving CSCs are able to reestablish the culture, indicating that they are responsible for therapy failure [55]. Chemotherapy-resistant CD133+ CSCs were also observed in lung and liver cancer [56, 57]. Similarly, the phenomenon of CSCs escape from therapy was also observed in xenograft studies. Chemotherapy treatment of xenotransplanted CSCs leads to an increase in CD133+ CSCs in the tumor [58]. This showed that CD133+ CSCs are more resistant to chemotherapy drugs in vivo compared to differentiated CD133+ cells. And breast CSCs and GBM CSCs isolated from patient specimens have showed selective resistance to a variety of chemotherapies [59, 60].

**Figure 1:**
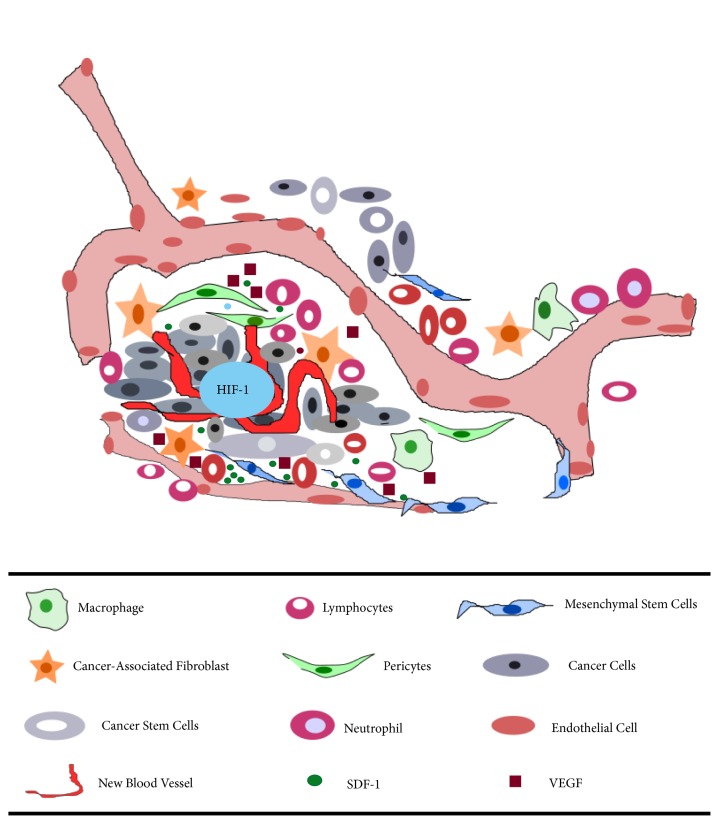
*The primary tumor microenvironment*. Cancer cells in primary tumors are surrounded by a complex microenvironment that consists of numerous cells, including endothelial cells of the blood vessel, cancer-associated fibroblast, lymphocytes, neutrophil, MSCs, macrophages, cancer stem cells, and pericytes. Solid tumors contain a significant fraction of microregions that are chronically or transiently hypoxic, in which HIF-1 associated signaling pathway is activated, thus inducing the expression of various downstream genes, including VEGF and SDF-1.

Furthermore, various studies have shown that the tumor stroma plays important roles in the survival, growth, and metastatic progression of cancer. In the hypoxic environment, the tumor stroma can increase its secretion of signaling proteins such as tumor necrosis factor-*α* (TNF-*α*), TGF-*β*, PDGF, and hepatocyte growth factor (HGF) [61]. In the meanwhile, tumor oxygenation status is tightly associated with its aggressive behavior. Experimental solid tumors contain a significant fraction of microregions that are chronically or transiently hypoxic. Hypoxia plays critical roles in tumor progression including tumor angiogenesis, mutation rate, metastasis and resistance to radiation and chemotherapy [62]. Many molecular pathways have been demonstrated to mediate these hypoxia-induced responses in tumors. Among them, hypoxia-inducible factor-1 (HIF-1) is a key signaling pathway regulating tumor responses to hypoxia [63]. Transiently hypoxic microenvironment in solid tumor may represent the stem cell niche to some extent, in which HIF-1*α* stabilization and activation of stromal-cell derived factor-1 (SDF1), VEGF, and Chemokine (C-X-C motif) Receptor 4 (CXCR4) occur, attracting MSCs homing and recruitment consequently [64, 65]. Furthermore, the state of tumor-induced hypoxia, which often perpetuates the inflammatory state, induces the secretion of numerous growth factors (e.g., endothelial growth factor-A, and fibroblast growth factor), thereby inducing MSCs recruitment and tumor growth through stimulation of tumor angiogenesis [23, 66]. The cancer microenvironment, MSCs, and CSCs are illustrated in Figure 1.

## 3. MSCs and Antitumor Therapy

### 3.1. MSCs-Derived Exosomes as Vehicles for Antitumor Therapy

Exosomes are nano-sized (<100 nm) and lipid-bilayer-enclosed extracellular vesicles that are released by many types of cells. They are found to play critical roles in intercellular communication via the transfer of genetic molecules such as mRNA and microRNAs, as well as proteins [67]. A common characteristic of human cancers is the aberrant expressions of either oncogenes, oncomiRs, or tumor suppressors. The MSCs-derived exosomes which contain a variety of miRNAs can be taken up by cancer cells and function in them. For example, miR-100 has been found to be downregulated in all subtypes of breast cancer, including the luminal A, luminal B, basal-like, and human epidermal growth factor receptor 2 (HER2) subtypes [34]. It is enriched in MSCs-derived exosomes and could suppress in vitro angiogenesis through modulating the mTOR/HIF-1*α*/VEGF signaling axis in breast cancer cells [35].

However, to date, studies with controversial outcomes on MSC-derived exosomes in tumor progression have been reported, including promoted effects [36–38] and suppressive effects [68, 69], as summarized in Table 3. The controversy effects of MSCs-derived exosomes may result from different tissue-derived MSCs used and different component of exosomes applied, different protocols applied for exosome collection, as well as different cancer model and stages of cancer studied. In addition, there is also another issue that the exosomes secreted by MSCs are not created equal. Thus, comprehensive studies are required to advance our knowledge and concerns over cancer research and treatment using MSCs-derived exosomes. One possible approach for clinical application of MSCs-derived exosomes for cancer treatment is that MSCs should be genetically engineered for stable expression of some cancer killer genes before the isolation of exosomes from MSCs, just as Sueon Kim et al. reported for generating antigen-specific CD8+ T cells for adoptive cell therapies against viral infection and tumors [70].

### 3.2. MSCs as Vehicles for Antitumor Therapy

MSCs have the characteristics of tumor tendency and avoidance of immune clearance; thus, it is promising that MSCs are utilized as vehicles to deliver anticancer treatments [71]. It has been demonstrated in a number of preclinical in vitro migration experiments and in various tumor models, such as hepatoma [72], leukemia cells [73], breast cancer [52], and osteosarcoma [74]. It may be an appropriate strategy that MSCs carrying anticancer drugs targeted treatment of tumors. For example, Bonomi et al. observed that MSCs-Paclitaxel (PTX) inhibit the proliferation of human myeloma cells in vitro 3D dynamic culture system [75]. The anticancer effect of MSCs-PTX has also been shown on pancreatic carcinoma cells in vitro [76]. MSCs are also promising tool for cisplatin (CDDP) delivery towards the tumor [71]. In addition, researches have shown that MSCs with suicide genes or apoptotic genes targeting for tumor is a promising approach. In vivo and in vitro studies have shown that the expression of interferon-*γ* in MSCs transfected by adenovirus can effectively kill glioma cells [77]. It is worth noting that in a model of lung metastasis of prostate cancer, MSCs expressing IFN-*β* could prolong the survival period, and its possible mechanism is that IFN-*β* could promote tumor cell apoptosis, inhibit angiogenesis, and increase the activity of natural killer cells [78]. Similarly, adenovirus-transfected MSCs expressing interferon-*γ* inhibit proliferation of leukemia cells and induce apoptosis of leukemia cells in vitro [79]. In models of lung metastatic carcinoma, a study has found that MSCs carrying TNF-related apoptosis-inducing ligand (TRAIL) reduce tumor growth and recurrence and inhibit the growth of lung metastatic foci in most mice [80]. Study has reported that in glioma mice, tumor tropism of umbilical cord MSCs carrying TRAIL was enhanced after irradiation and its proapoptotic effect on tumor cells was enhanced by MSCs-TRAIL [81]. In addition, the previous and our studies also have demonstrated that MSCs could be genetically modified with herpes simplex virus thymidine kinase (HSV-TK), and the cancer cells could be killed by HSV-TK/GCV suicide gene therapy [82–84]. A recent study showed that histone deacetylase inhibitors (HDACis) induced apoptosis of chemoresistant cells effectively, like CD123/CD47-positive cells, which were found as maybe serving as a key role for chemoresistance in tumor microenvironment. Furthermore, HDACis efficiently targeted and removed chemoresistant leukemia blasts in a xenograft AML mouse model [85].

**Figure 2:**
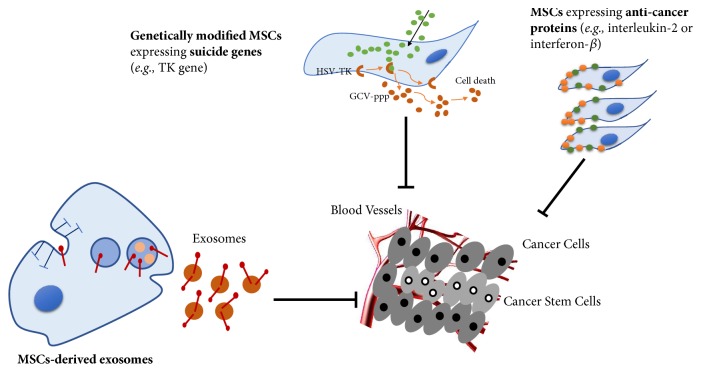
*Strategies of targeted-cancer therapy*. MSCs have been utilized as vehicles to deliver anticancer treatments due to their tumor-tropic property. Genetically modified MSCs expressing suicide genes (e.g., TK gene) have been used to treat cancer effectively in vitro and in vivo through inducing cell death. Additionally, MSCs can be induced to express anticancer proteins (e.g., IL-2 or IFN-*β*), to generate prodrug activating enzymes or to deliver oncolytic viruses and the active drug for tumor-targeting. Simultaneously, MSCs-derived exosomes also suppressed tumor growth.

The immune system plays an important role in monitoring the growth of malignant cells. Therefore, stimulating the body's own immune system for antitumor treatment is a highly promising strategy. Interleukins (ILs) are cytokines that regulate inflammation and immune response and have been shown to exhibit antitumor effects through direct tumor-killing or active regulation of the endogenous immune system [86]. MSCs have been utilized to deliver interleukins that can improve the anticancer immune surveillance by activating NK cells and cytotoxic lymphocytes [86]. For example, the IL-18 secreted MSCs were correlative with enhanced T cell infiltration and antitumor immunity in mice bearing invasive and noninvasive gliomas [87]. Similarly, MSCs engineered to express IL-12 prevented metastasis into the lymph nodes and other internal organs as well as increased tumor cell apoptosis in mice bearing preestablished metastases of melanoma, breast, and hepatoma tumors [88]. Also, MSCs engineered to express IL-12 was tested in different mouse tumor models of melanoma and glioma [46]. Other immune-stimulatory molecules, like CX3C chemokine fractalkine (CX3CL), have also been engineered in MSCs. CX3CL1 is known as a strong T cell chemoattractant. Recent studies reveal that CX3CL1 is a driver of T cell migration to the omentum in esophagogastric adenocarcinoma (EAC). Previous research has shown that injection of an adenoviral vector expressing CX3CL1 can induce strong antitumor immune responses by activating both NK cells and T cells [89]. Similarly, intravenous or intratracheal delivery of MSCs-CX3CL1, activating T cells and NK cells, was observed to strongly inhibit process of lung metastasis and increase survival of mice carrying lung metastases cells [90, 91]. Taken together, the tumor-trophic homing capacity makes MSCs ideal cellular delivery vehicles for personalized cell-based targeted-cancer gene therapy. And the strategies of targeted-cancer therapy were summarized in Figure 2.

### 3.3. Inhibition of Migration for Antitumor Therapy

Tumor has been seen as a “wound that never heals” which enrolls MSCs in its microenvironment through production of paracrine and endocrine signals. So theoretically, inhibition of MSCs homing will prevent the growth of tumor. For instance, PDGF receptor *β* (PDGFR*β*) has been reported to play an important role in recruitment of MSCs towards tumor sites [92]. And Simona Camorani et al. have demonstrated that interfere with the PDGFR*β*-mediated crosstalk between BM-MSCs and tumor cells using a nuclease-resistant RNA aptamer could inhibit the migration of MSCs towards tumor cells and hampering tumor aggressiveness [93]. The classic signaling governing MSCs homing is SDF1-CXCR4 axis. SDF1 is highly expressed in active multiple myeloma, as well as in bone marrow sites of tumor metastasis, neutralizing SDF1 with a high-affinity L-RNA Spiegelmer to SDF-1 has been demonstrated to diminish the disease progression [94]. In tumor biology, a number of studies observed the requirement of Akt and Wnt signaling for the migration, invasion, and survival of tumor cells [95–97]. Recent studies have shown that MSCs are involved in mediating these signaling pathways to influence migration of tumors. In glioma cells and in the nude mice tumors, upregulation of PTEN by hUCBSC downregulated Akt and (phosphoinositide 3-kinase) PI3K signaling pathway results in the inhibition of migration [98]. Similarly, results of a study demonstrated that overexpression of HNF4*α* suppresses HCC progression by reducing hepatoma cell growth and metastasis through downregulating the Wnt/*β*-catenin signaling pathway [99].

## 4. Clinical Trials of MSCs in Cancer Therapy

A small number of registered clinical trials for the treatment of solid tumors with MSCs are underway. These trials have been inspired by successful preclinical trials, although some results have not been published yet. The first clinical trial of gastrointestinal tumors worldwide utilizing genetically engineered MSCs in humans has been reported (TREATME1) [100]; this trial uses MSCs-delivery of HSV-TK under the control of the CCL5 promoter. This is a successful phase I/II clinical trial. Another two registered clinical trials with MSCs have primarily focused on ovarian cancer. One of them is a phase I clinical trial sponsored by M.D. Anderson Cancer Center, in which human MSCs transfected with interferon beta (MSCs-IFN*β*). The purposes of this clinical trial are to test the safety of MSCs-IFN*β* and to find the highest tolerable dose of human MSCs-IFN*β* that can be given to patients with ovarian cancer. Similarly, Mayo Clinic initiated a phase I/II trial to find the side effects and best dose of MSCs infected with oncolytic measles virus encoding NIS (MV-NIS) and to observe its effect on patients with ovarian cancer. In a clinical approach for the treatment of lung cancer, allogeneic MSCs expressing a full length version of TRAIL have been used. MSCs as gene-therapeutic vehicle aim to deliver the TRAIL. In addition, one clinical trial for treating liver cancer with MSCs is on the registry and is recruiting subjects. The purpose of this trial was to study whether MSCs may influence the outcome of graft versus host response in liver transplantation of liver cancer patients. In a phase I clinical test, allogeneic bone marrow-derived MSCs were infused in men with localized prostate cancer [101]. The primary objective was to assess safety and cancer-homing ability of MSCs. However, in this study, MSCs did not home primary tumors in sufficient levels to kill cancer cells or inhibit tumor growth. Although the treatment results have not been published or have not achieved the expected objectives, more attention and patience are needed to promote the clinical transformation of MSCs in tumor treatment. In summary, MSCs and their secreted exosomes have great potential for tumor therapy. Meanwhile, to accelerate the transformation from preclinical research to clinical application, more efficacy and safety of these therapeutic approaches need to be provided by preclinical studies.

## 5. Conclusions and Prospective

This article primarily discusses the recent progress of the complex roles of multipotent MSCs in tumor microenvironment, progression, and potential clinical applications. The function of CSCs in tumor microenvironment should be paid more attention to, which is critical for development of cancer cells. The roles of miRNAs and signaling pathways in tumor microenvironment need to be intensively studied, which may provide us with new means to accurately treat cancer. We should also pay more attention to the molecular mechanism of antitumorogenic activity of MSCs, which may improve the precision of targeted therapy. Importantly, reducing the growth stimulation and malignant transformation of MSCs in tumor targeted therapy will accelerate clinical transformation.

## Figures and Tables

**Table 1:** Favoring effect of MSCs on tumors.

Author	MSC origin	Tumor model	MSC: tumor cell ratio	Outcomes	Mechanisms
Chaturvedi P et al. [17]	Human bone marrow-derived MSCs	Breast( MDA-MB-231, MDA-MB-43)	1:1 coinjection	Increased metastasis	activation of the hypoxia-inducible factors (HIFs)

Walter, M. et al. [21]	Human adipose stromal cells (ASCs)	Human breast cancer cell line MCF-7	1:1 coinjection	Increased migration and invasion	Secretion of IL-6

Tsai, Kuo–Shu et al. [22]	Human bone marrow-derived MSCs	Human colorectal cancer cell line HT-29	1:100 coinjection	Promoted tumor sphere formation and tumor initiation	IL-6 secreted by MSCs signaled through STAT3

Zhang, Ting et al. [23]	Human fetal bone marrow stem cells (hBM-MSCs)	4T1 mouse mammary tumor cell line	1:1 coinjection	Increased tumor growth	Neovascularization (secretion of macrophage inflammatory protein-2, vascular endothelial growth factor, transforming growth factor-beta and IL-6)

El-Haibi, Christelle P. et al. [24]	Human bone marrow-derived MSCs	MDA-MB-231 and MCF7/Ras breast cancer cells	1:1 coinjection	Enhanced metastasis	Increased de novo production of lysyl oxidase (LOX)

Patel, Shyam A. et al. [25]	Human bone marrow-derived MSCs	Highly aggressive MDA-MB-231 breast adenocarcinoma, low-invasive MCF-7 breast adenocarcinoma, T47D breast adenocarcinoma, P815 murine mastocytoma	1:1 (T47D and MSCs 2 × 10^5^ /ml each) were added in 500 *μ*l volumes to attain a 50:1 ratio of mononuclear fractions (PBMC)/MSC and PBMC/T47D	Protected breast cancer cells from immune clearance	Through Tregs, inhibited NK cell and CTL functions

Chandler, Emily M. et al. [26]	Human adipose-derived stem cells (ADSCs)	MCF-7 and MDA-MB-231	1:1 co-injection	Promoted tumorigenesis and angiogenesis	Bidirectional signaling; ADSCs differentiated into cancer-associated myofibroblasts

Gonzalez, Maria E. et al. [27]	Human breast cancer metastatic sites-derived MSCs	Breast cancer cell lines MDA-MB-231, MCF7, and MDA-MB-436	MSCs were orthotopically injected into the mammary fat pads (1 × 10^6^ cells/mouse)	Loss of DDR2 in MSCs impaired their ability to promote DDR2 phosphorylation in BC cells, as well as BC cell alignment, migration, and metastasis	Reduced migration and metastasis

**Table 2:** Inhibitory effect of MSCs on tumors.

Author	MSC origin	Tumor model	MSC: tumor cell ratio	Outcomes	Mechanisms
Ho, Ivy AW et al. [28]	Human bone marrow-derived MSCs	Primary human glioma cells	1:1 (coinjection)	Reduction in tumor volume and vascular density	Secretion of soluble factors inhibiting endothelial progenitor cells recruitment and impaired tumor angiogenesis

Leng, Liang et al. [29]	Human umbilical cord-derived MSCs	Human breast cancer cell line MDA-MB-231	1:1 (injection of MDA-MB-231 first, injection of MSCs 13 days later)	Antitumor effect	Inhibited tumor angiogenesis and induced cell apoptosis

Meleshina, Aleksandra V. et al. [30]	Human bone marrow-derived MSCs	MDA-MB-231 human breast adenocarcinoma cell line	1:1 (MDAMB-231-Turbo FP650 cells injection fist, injection of MSCs 10 days later)	Suppressed tumor growth and lung metastasis	Reduced proliferative activity of cancer cells

Dasari, Venkata Ramesh et al. [31]	Human umbilical cord blood-derived MSCs	Two high-grade human glioma cell lines (SNB19 and U251) and two xenograft cell lines (4910 and 5310)	1:4 (MSCs injection 7 days after tumor implantation)	Inhibited tumor growth	Upregulation of phosphatase and tensin homolog deleted on chromosome 10 (PTEN) in tumors induced cellular death through decreasing XIAP expression

Xie, Chan et al. [32]	Human bone marrow-derived MSCs (interferon beta (IFN-*β*) modified)	HCC cell lines HepG2 and Huh7	300:1 (MSC injection 3 days after HCC inoculation)	Inhibition of HCC proliferation	Inhibition of AKT/FOCO3a pathway

Wu, Ning et al. [33]	Human umbilical cords-derived MSCs (transfection of hepatocyte nuclear factor 4*α* (HNF4*α*)	Liver cancer cell lines HepG2 and SK-Hep-1	1:5 (MSC injection 24 h after tumor implantation)	Inhibited HCC proliferation and invasion	Downregulation of Wnt/*β*-catenin signaling in HCC cells

François, Sabine et al. [20]	MSCs from human or rat bone marrow	Colorectal cancer cell lines (HT29, HCT-116, LS513, and CC531)	N/A	Attenuation of tumor progression	Modulation of immune component

**Table 3:** Effects of MSCs-derived exosomes on tumors.

Author	Exosome origin	Tumor model	Outcomes	Mechanisms
Li, Hongdan et al. [34]	Human bone marrow MSCs from patients undergoing hip-replacement surgery	Colon cancer cells (HCT-116, HT-29, and SW-480)	Increased the population of colon cancer stem cells	miR-142-3p in exosomes promoted the Notch signaling pathways by downregulating Numb

Zhang, Yanling et al. [35]	Human omental adipose-derived MSCs from cancer-free female donors	Human EOC cell lines (SKOV3, A2780, and HO-8910)	Promoted cancer progression	Affect proteomic profile of tumor cells via paracrine mechanism

Roccaro AM et al. [36]	Human bone marrow MSCs from normal or cancer patients	Multiple myeloma (MM) cells	MM BM-MSCs–derived exosomes promoted MM tumor growth, normal BM-MSC exosomes inhibited the growth of MM cells	Impact MM cell adhesion

Makiko Ono et al. [37]	Human bone marrow MSCs	BM2 cells	Slowed tumor growth	Exosomal transfer of miR-23b and its suppression of MARCKS

Reza AM et al. [38]	Human adipose MSCs	A2780 and SKOV-3 ovarian cancer cells	Inhibited proliferation of ovarian cancer cells	Upregulates proapoptotic molecules

## Abbreviations

ADSCs: Adipose-derived stem cells
ASCs: Adipose stromal cells
BC: Breast cancer
CSCs: Cancer stem cells
CXCR4: Chemokine (C-X-C motif) Receptor type 4
CTL: Cytotoxic T lymphocyte
CX3CL: CX3C chemokine fractalkine
EAC: Esophagogastric adenocarcinoma
hBM-MSCs: Human fetal bone marrow stem cells
HCC: Hepatocellular carcinoma
HER2: Growth factor receptor 2
HGF: Hepatocyte growth factor
HIFs: Hypoxia-inducible factors
hUCMSCs: Human umbilical cord MSCs
HSV-TK: Herpes simplex virus thymidine kinase
hUCMSC-EVs: hUCMSC-derived extracellular vesicles
IL-6: Interleukin-6
IFN-*β*: Interferon beta
LUAD: Lung adenocarcinoma
LOX: Lysyl oxidase
MSCs: Mesenchymal stem cells
NK cells: Natural killer cells
PDGF: Platelet-derived growth factor
PI3K: Phosphoinositide 3-kinase
PDGFR*β*: PDGF receptor *β*
PTEN: Phosphatase and tensin homolog deleted on chromosome 10
PTX: Paclitaxel
SDF1: Stromal-cell derived factor-1
TGF-*β*: Transforming growth factor-*β*
TNF-*α*: Tumor necrosis factor-*α*
TRAIL: TNF-related apoptosis-inducing ligand
VEGF: Vascular endothelial growth factor.
